# Interindividual variability and individual stability of pain- and touch-related neuronal gamma oscillations

**DOI:** 10.1152/jn.00530.2021

**Published:** 2023-04-05

**Authors:** Elia Valentini, Alina Shindy, Viktor Witkovsky, Anne Stankewitz, Enrico Schulz

**Affiliations:** ^1^Department of Psychology and Centre for Brain Science, University of Essex, Colchester, United Kingdom; ^2^Department of Neurology, University Hospital, LMU Munich, Munich, Germany; ^3^Department of Theoretical Methods, Institute of Measurement Science, Slovak Academy of Sciences, Bratislava, Slovak Republic; ^4^Department of Neuroradiology, Technische Universität München, Munich, Germany; ^5^Department of Radiology, University Hospital, LMU Munich, Munich, Germany; ^6^Department of Medical Psychology, Ludwig-Maximilians-Universität München, Munich, Germany

**Keywords:** EEG, gamma oscillations, pain, stability, variability

## Abstract

Brief painful laser and innocuous tactile stimuli have been associated with an increase of neuronal oscillations in the gamma range. Although it is indicated that event-related gamma oscillations may be highly variable across individuals, to date no study has systematically investigated interindividual variability and individual stability of induced gamma synchronization. Here, we addressed this question using two EEG datasets. The first dataset contains two repeated sessions of tactile and painful stimulation from 22 participants. The second dataset contains a single session of painful stimulation from 48 participants. In the first dataset, we observed gamma responses in the majority of the included participants. We found a broad interindividual variety of gamma magnitudes, time-frequency (TF) response patterns, and scalp topographies. Some participants showed a gamma response with individually unique time-frequency patterns, others did not exhibit any gamma response. This was reproducible and therefore stable; subjects with a large gamma magnitude in the first session showed a large gamma magnitude and a similar response pattern in the follow-up session. The second dataset confirmed the large between-subject variability, but only a fraction of the included participants exhibited laser-induced gamma synchronization. Our results indicate that current EEG measures do not reflect the complex reality of the diverse individual response patterns to brief pain and touch experiences. The present findings question whether a similar phenomenon would be observed in other neuroscience domains. Group results may be replicable, but could be driven by a subgroup of the sample.

**NEW & NOTEWORTHY** The interpretation of gamma activity in response to noxious and innocuous somatosensory stimuli has sparked controversy. Here, we show that participants’ gamma oscillations measured through electroencephalography vary. Although some participants do not show a distinct gamma response, others exhibit stable and reliable response patterns in terms of time, frequency, and magnitude.

## Graphical abstract

**Figure:**
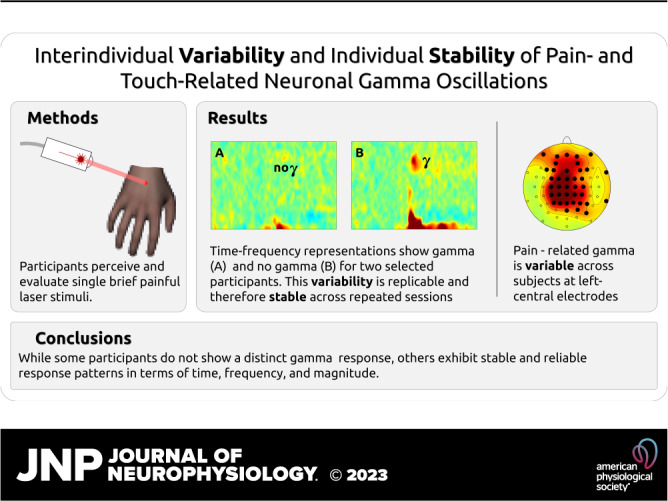

## INTRODUCTION

Research spanning several decades has highlighted the crucial role of high-frequency brain oscillations in perceptual integration (1, 2). A number of these publications focused on the investigation of pain-related and touch-related neuronal oscillations in the gamma range (3–7). There are fewer studies on gamma responses to brief touch (5) and more extended vibrotactile stimulation (8). The majority of studies showed an increase of gamma activity in response to brief phasic pain (9–13), long-lasting pain (6, 14, 15), and from intracerebral recordings of the insular cortex (16, 17).

However, studies differed in their interpretations of the importance of gamma oscillations on perception. Most studies report the role of gamma on the perception of pain without excluding the potential contribution of other cortical oscillatory processes (11, 17). Other studies suggested a particularly close relationship between gamma and pain perception (18) or claimed that gamma oscillations are the only neuronal oscillation predictive of pain perception (19). In contrast, there are doubts about the neuronal origin of gamma oscillations in response to noxious stimuli (20). Two potential reasons may explain the opposing perspectives.

### Insufficient Artifact Cleaning

First, EEG responses to painful laser stimuli in the gamma range are prone to event-related muscle artifacts, i.e., from saccades, face, and neck muscles (21–24). The comparison of various approaches to tackle muscle artifacts underlined the importance of data quality (25, 26). Consequently, studies that do not sufficiently clear the data of artifacts will inevitably misinterpret high-frequency artifacts as cortical signals (24) or vice versa (20, 27). This issue can be mitigated with the application of algorithms for the identification and removal of high-frequency EEG features of extracortical origin (26, 28, 29). As many of these approaches are using source separation methods (25, 26, 28), the use of a multielectrode array is imperative to provide sufficient independent input. In addition, high-density coverage of the scalp helps to assess the plausibility of the neuronal effect by evaluating gamma topographies (22).

The low signal-to-noise ratio of the data at higher frequency spectra and the diversity of artifacts of extracortical origin may not be sufficiently addressed with fully automated approaches of artifact removal. A single-trial check of time-frequency (TF)-transformed data (preferably decomposed with independent component analysis, ICA) is of utmost importance to ensure the best possible data quality (5, 17). We thus recommend expert scrutiny and assessment of the decomposition output (22, 27). Anything below these standards can raise legitimate doubts about the cortical origin of the gamma response (24).

### Interindividual Variable Response Patterns and Absence of Gamma Oscillations

Second, some researchers may have failed to detect gamma oscillations in response to painful laser stimulation. Although gamma effects at group level have been repeatedly published (11, 16), individual data show that not every participant has a clear gamma response. Indeed, the number of subjects per study that exhibit gamma oscillations can vary substantially; Tiemann et al. (30, unpublished aspects of the study series) could not identify any significant gamma response in a sample of 40 patients with fibromyalgia and age-matched controls. Consequently, studies may exhibit effects of sample variability as suggested by studies reporting laser-induced gamma effects at variable frequencies (5, 30–33), as broad-band effects (19, 34), and at different latencies (3, 35, 36). This variability also applies to studies on long-lasting pain, which reported a positive relationship between the magnitude of gamma and pain intensity at group level for frontocentral electrodes (6, 14). These studies also show the variability of individual pain-related gamma oscillations by reporting single-subject results with no relationships or even negative relationships between gamma activity and pain intensity for some participants (6, 14).

Both problems entail elements of cultural and systemic tradition in the field. We focus on the second aspect and argue that research on the neurophysiology of pain will greatly benefit from a more in-depth assessment of individual data. Here, we explore the interindividual variation of event-related gamma oscillations and the individual stability of event-related gamma across subjects in repeated recordings. We hypothesize that participants with a clear gamma response would exhibit gamma in either session of painful and tactile stimulation. Relatedly, subjects with low or no gamma should exhibit this effect in both sessions.

## METHODS

### Subjects

All participants gave written informed consent. The studies were approved by the local ethics committees and conducted in conformity with the Declaration of Helsinki.

#### Dataset 1.

We used data previously reported in two distinct studies (5, 37). EEG was recorded from 22 healthy male human subjects with a mean age of 24 yr (21–31 yr).

#### Dataset 2.

We used data previously reported by Nickel et al. (38), which are available online at the Open Science Foundation (https://osf.io/jw8rv/). The dataset contains EEG recordings of 48 healthy human subjects (25 males) with a mean age of 24 yr (18–37 yr).

### Paradigm

#### Dataset 1.

The participants attended two experimental sessions with a time interval of 2 wk. Within each session, a total of 75 painful cutaneous laser stimuli and 75 touch stimuli of matched intensities were delivered to the dorsum of the right hand; tactile and painful stimuli were separately delivered in two counterbalanced blocks. The laser device used was a Tm:YAG laser (StarMedTec GmbH, Starnberg, Germany) with a wavelength of 1,960 nm, a pulse duration of 1 ms, and a spot diameter of 5 mm. The physical energy of the painful stimulation was kept constant at 600 mJ. Tactile stimuli with a force of 181 mN were applied using von Frey monofilaments delivered through a computer-controlled device as described in detail previously (5, 39).

The interstimulus interval (ISI) for both modalities was randomly varied between 8 and 12 s. Participants perceived the stimuli with closed eyes. Three seconds after each stimulus, they were prompted by an auditory cue to verbally rate the perceived intensity of the stimulus on a 0–10 numerical rating scale. For painful stimuli, this was anchored by no pain (0) and maximum pain (10) the subjects were willing to tolerate during the experiment. For tactile stimuli, the scale ranged between no perception (0) and the strongest imaginable touch that was not perceived as painful (10). The entire procedure of dopamine depletion, which neither significantly affected the perception of pain intensity nor the magnitude of gamma oscillations, has been described elsewhere (37). The reanalysis of the current data involves new integrated statistical modeling as well as advanced handling of muscle artifacts (28).

#### Dataset 2.

The participants contributed to a paradigm on pain-related prediction errors and received 160 noxious radiant heat stimuli of two different intensities (80 high and 80 low), which were cued by visual stimuli. Stimulus intensity was set to 3.5 J for high-intensity stimuli and 3 J for low-intensity stimuli. The pain stimuli were applied 1.5 s after the offset of the cue. Three seconds after the delivery, participants were prompted to rate the perceived pain intensity on a numerical rating scale ranging from 0 (no pain) to 100 (maximum tolerable pain). The experiment had four runs of 40 trials, separated by 3 min. The laser device was an Nd:YAG laser (Stimul 1340, DEKA M.E.L.A. srl) with a wavelength of 1,340 nm, a pulse duration of 4 ms, and a spot diameter of ∼7 mm. The authors used high-intensity and low-intensity pain stimuli for the analysis of the data; for detailed descriptions of the experimental procedure see the study by Nickel et al. (38).

### EEG Recording and Preprocessing

#### Inclusion of two datasets.

From the two independent datasets, only *dataset 1* allows a complete analysis of all measures of variability and stability. The test on variability (*datasets 1* and *2*) quantifies whether there is a difference in the magnitude of experienced pain and the magnitude of pain-related gamma oscillations across subjects. The dual-session data (*dataset 1*) enabled us to investigate the stability of cortical effects across separate recording sessions. *Dataset 2* has been included to further validate our analysis of the between-subject variability of pain ratings and gamma magnitudes. This could be accomplished by analyzing the induced gamma in the ICA space. Therefore, no extensive data cleaning and back-transformation to EEG for *dataset 2* was needed. It is noteworthy that this dataset includes only a single session and does not permit any analysis of the stability between-session effects.

#### Dataset 1.

EEG data were recorded using an MRI-compatible electrode cap (FMS, Munich, Germany). The montage included 64 electrodes, was referenced to the FCz electrode, grounded at AFz, and sampled at 1 kHz with 0.1 µV resolution. Impedance was kept below 20 kΩ. Raw subject-wise concatenated EEG data, which included pain and touch sessions, were preprocessed in Brain Vision Analyzer software (Brain Products, Munich, Germany). After the application of a 0.5-Hz high-pass filter, data were decomposed into 64 components using ICA. Components related to horizontal and vertical eye movements, as well as major artifacts, were removed and data were back-transformed into EEG signals. A second ICA was conducted and muscle artifacts were removed from the components’ time courses using a dedicated algorithm (28) and epoched from −1,100 to 1,500 ms poststimulus. A few residual artifact components were removed in a third ICA (see *Data quality check*). After the retransformation to EEG, artifact-free data were downsampled to 512 Hz and rereferenced to the common average reference.

#### Dataset 2.

EEG data were recorded using an MRI-compatible electrode cap (FMS, Munich, Germany). The montage included 64 electrodes, was referenced to the FCz electrode, grounded at FPz, and sampled at 1 kHz with 0.1 µV resolution. Impedance was kept below 20 kΩ. Raw subject-wise concatenated EEG data from four subsessions were preprocessed in Brain Vision Analyzer software (Brain Products, Munich, Germany). After the application of a 0.5-Hz high-pass filter, data were decomposed into 64 components using ICA, downsampled to 512 Hz, and epoched from −1,100 to 1,500 ms poststimulus.

#### Time-frequency analyses.

Sixty-five channel EEG data (*dataset 1*) and 64 component ICA data (*datasets 1* and *2*) were exported to Matlab (R2020a; The MathWorks). Time-frequency decomposition was performed using custom programming on the basis of standard mathematical and signal analysis functions. We applied a single-trial Hanning tapered, short-time fast Fourier transformation (FFT). The moving window had a length of 200 ms, was padded with zeros to obtain a 1-s window length, and was shifted by two data points. The magnitudes of each frequency were computed from 1 Hz in steps of 1 Hz (interpolated) up to 100 Hz. On a single-trial basis, time-frequency representations (TFRs) were computed and transformed into percent signal change values with respect to the single-trial baseline averaged from −1,000 to −100 ms.

#### Data quality check.

Alongside the percent signal change transformations for statistical analysis, single trials were also z-transformed. This has been done separately for each frequency and across EEG electrodes and across components. TF plots of single-trial ICA and EEG data were checked visually for high-frequency artifacts. For *dataset 1*, we excluded 225 trials across the entire dataset (per subject, *pain session 1*: 3.2 ± 3.3, *pain session 2*: 2.1 ± 1.9, *touch session 1*: 2.7 ± 2.3, *touch session 2*: 2.1 ± 2.1). There was no difference regarding the deleted trials between sessions (pain: *t* = 1.26; *P* = 0.22; touch: *t* = 0.87, *P* = 0.4) or between stimulus modalities (*t* = 0.53, *P* = 0.6; all paired *t* tests).

#### Independent component-based analysis.

The statistical analyses based on IC data permitted us to specifically select individual gamma responses irrespective of the scalp topography. A subset of the participants (15/22 from *dataset 1*; 8/48 from *dataset 2*) exhibited one to four components with a clear gamma response. All components with gamma responses were always tied to a component with evoked activity, had a topography associable with cortical sources, and were artifact-free (no contamination with eye movements, saccades, or muscle spikes). In case a participant exhibited more than one component with a dedicated gamma effect (applies to *datasets 1* and *2*), we selected the component that contained the strongest gamma effect (defined as a greater % signal change relative to the baseline of this component). This approach has two advantages. First, we take individual variation into account. The present data show tremendous variability in gamma responses with distinct topographical distributions and unique but replicable TF ranges. Unlike data processing on EEG electrodes, a second advantage is that these components are largely artifact free.

The shape of the individual gamma TF windows was defined through *t* test for each TF datapoint in the gamma range (40–100 Hz, 0–500 ms) with its respective baseline (−1,000 to −100). Due to a large number of tests (60 frequency points × 256 time points), data were individually corrected for multiple testing. The individual thresholds were defined by randomizing baseline and poststimulus data 5,000 times (see *Correction for multiple testing*). From these thresholded time-frequency windows, averaged gamma signal change magnitudes were computed for each trial. For subjects without gamma components, we selected the component with the largest evoked response. For these subjects without significant gamma, magnitudes were extracted for every single trial by averaging the signal change data from the literature-based TF window (76–86 Hz; 0.15–0.35 s; 5, 13).

#### Electrode-based analysis.

EEG-space analyses on *dataset 1* were included to allow the comparison of the gamma topographies with previous studies. Statistical analyses were conducted separately for each electrode. Based on our previous findings, we defined the gamma windows for the analysis of EEG electrodes by averaging the signal change data from the literature-based TF window. Gamma responses were computed as percent signal change in reference to the prestimulus baseline (−1,000 to −100 ms) for each trial and frequency. We computed the signal change for each TF data point before averaging within the predefined literature-based gamma window.

### Statistical Analysis

#### Individual variability of perception and cortical processing.

For both datasets, we quantified the differences in the experienced pain/touch and the apparent differences in the occurrence of gamma oscillations (see single-subject TF maps). To take the entire data structure into account, we analyzed the data at single-trial level. Consequently, we computed linear regressions on *1*) behavioral data (ratings; *datasets 1* and *2*), *2*) gamma responses from ICA components (*datasets 1* and *2*), and *3*) gamma responses from EEG electrodes (*data set 1*) by fitting the data using the “fitlm” function in Matlab:

(*1*) dependent_vars∼subject

*F* statistics were calculated by subsequent ANOVAs.

We tested whether the across-subjects variability in ratings and gamma magnitudes is stable across sessions. We separately ran the statistical analyses for both pain and touch. All tests were performed on three dependent variables: *1*) behavioral data, *2*) gamma magnitudes from ICA components (*datasets 1* and *2*), and *3*) gamma magnitudes from EEG electrodes (*dataset 1*). The analysis of stability has three different aspects, which provide complementary information on the stability of laser-induced gamma oscillations and ratings across sessions: the ranking of participants, the potential change between sessions, and the similarity of time-frequency patterns.

#### Stability—preserved ranking of participants.

A high stability across sessions means that subjects with a high behavioral rating or strong gamma magnitude in the first session would also exhibit a strong effect in the second session: the participants’ ranking would be preserved. We computed Kendall’s τ coefficient on pairs (*session 1*, *session 2*) of averaged ratings and gamma magnitudes:

(*2*) $$\text{τ}=\frac{\left(\text{number of concordant pairs})-(\text{number of discordant pairs}\right)}{\left(\begin{array}{c} n\\ 2 \end{array}\right)}$$

#### Stability—systematic change between sessions.

A high stability across sessions would also imply that there is no systematic change (ratings, gamma) from the first to the second session, e.g., a drop of pain perception or gamma magnitude in the second session compared with the first session. This has been analyzed by modeling the dependency of the three dependent variables (ratings, ICA-gamma, EEG-gamma) in regard to the order of testing sessions (*variable session*) or the depletion intervention (*variable d_depletion*). Linear mixed-effects models (LMEs; 40) provide fixed-effects parameter estimates for each subject and session.

(*3*) dependent_vars∼d_depletion+session+subject+(1|subject:session)

*F* statistics were calculated on the parameter estimates by subsequent ANOVAs.

#### Stability—similarity of time-frequency patterns across sessions.

We further explored the stability of individual gamma TF patterns by testing whether the extent of the gamma TF pattern in the first session matches the gamma TF pattern in the second session. We computed datapoint-by-datapoint Pearson correlations between the first and second session for the selected components within the gamma range (40–100 Hz, 0–500 ms). The correlation results in a high coefficient if corresponding TF coordinates show similarly high or low values. The results yield a correlation coefficient for each participant. A paired *t* test compares the within-subject correlation between sessions with the correlation for the subjects’ first recordings with the averaged correlations of the second recordings of the other subjects. Correlation coefficients were Fisher z-transformed before *t* test and averaging.

#### Correction for multiple testing.

To correct for multiple testing for the analyses on the multivariate EEG data, we applied a randomization approach (function “randperm” in Matlab). Depending on the variable, data were shuffled and analyses were repeated 5,000 times. For the analysis on the variability across subjects, the single trials were randomly assigned to the participants (e.g., 5,000 times randperm(3161) for pain in *dataset 1*). For the effect of depletion and session, we randomized the EEG gamma data between sessions but within subjects [randperm(150)]. For the correlation between sessions, we randomized the gamma data of the first session between subjects [randperm(22)].

The highest absolute τ-values or *F* values of each repetition across all electrodes were extracted. Each of these procedures resulted in a right-skewed distribution of the 5,000 absolute max values for each analysis (see Supplemental Material). Based on these distributions, the statistical thresholds were determined using the “palm_datapval” function publicly available in PALM (https://fsl.fmrib.ox.ac.uk/fsl/fslwiki/PALM; 41). For all tests on multiple comparisons, we applied a statistical threshold of *P* < 0.05.

## RESULTS

### Descriptive Statistics

#### Behavioral data.

**Figure 1.**
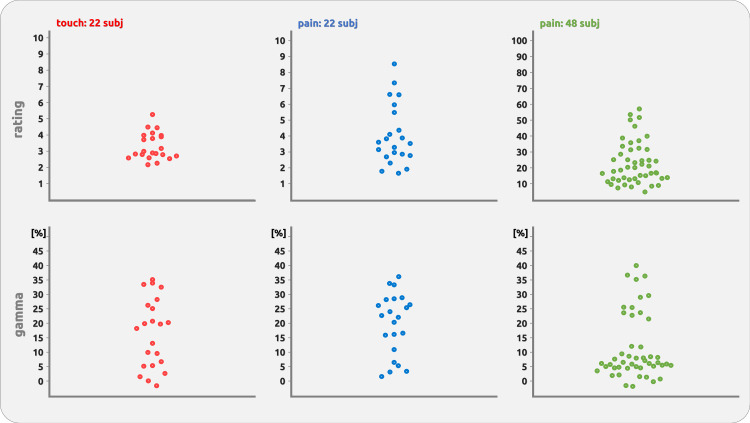
Bee plots of individual variability of ratings and gamma. The bee plots show remarkable variability across subjects despite identical stimulus physical properties.

For *dataset 1*, all participants received stimuli with the same physical intensity. The rating across all sessions and subjects was 4.06 ± 1.93 for pain and 3.31 ± 0.91 for touch (Fig. 1, *A* and *B*, means ± STD, scale ranging from 0 to 10). For *dataset 2*, participants received two levels of pain intensity with an across-subjects average of pain ratings of 13 ± 10 for low-pain trials and 33 ± 19 (Fig. 1*C*) for high-pain trials (scale ranging from 0 to 100).

#### Gamma data.

**Figure 2.**
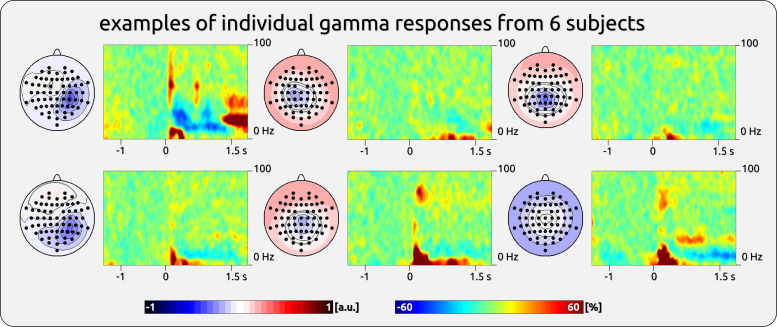
Example of gamma variability in single-subject components. Examples were taken from *study 2*. *Left*: topographies of independent component analysis (ICA) components. The topographies were normalized and are ranging from −1 to 1 (arbitrary units: a.u.). *Right*: time-frequency representation (TFR) plots.

**Figure 3.**
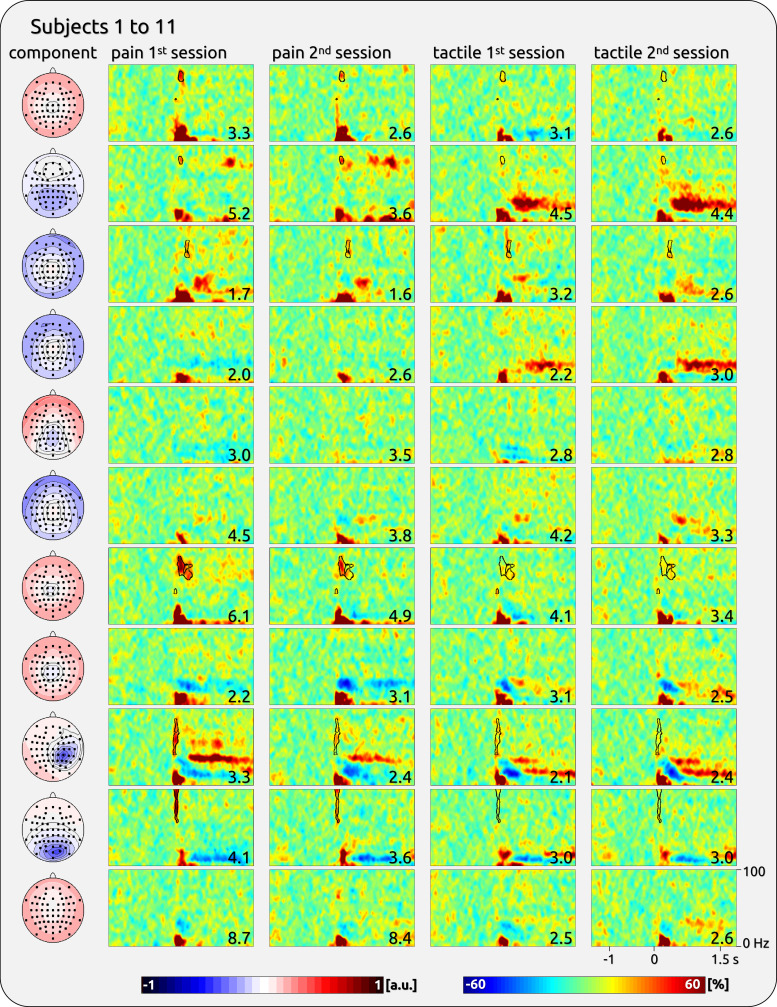
Single-subject components with gamma response from the first half of the participants of *data set 1*. *Left*: topographies of independent component analysis (ICA) components. *Right*: time-frequency (TF) representation (TFR) separately for the first and second sessions and for pain and touch. The outlined TF areas were selected to estimate the stability and variability of the gamma magnitude. The *bottom right* corner of each spectral plot contains the individual’s averaged rating of the sensory experience (scale 0 to 10). The topographies were normalized and are ranging from −1 to 1 (arbitrary units: a.u.).

**Figure 4.**
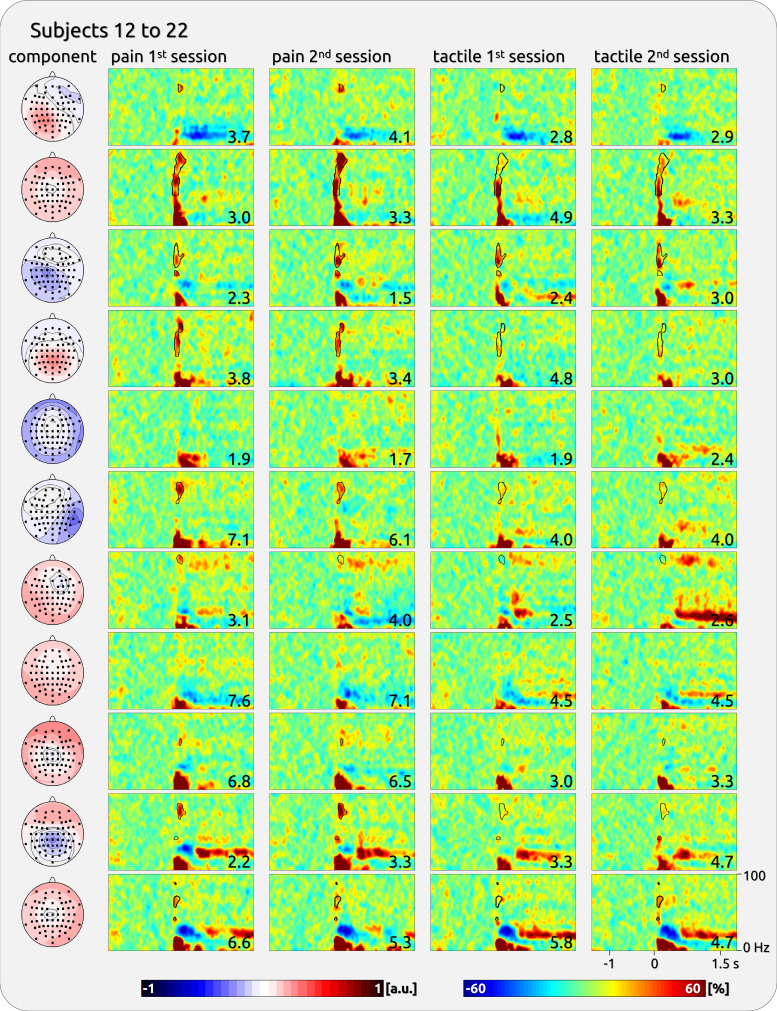
Single-subject components with gamma response from the second half of the participants of *dataset 1*. *Left*: topographies of independent component analysis (ICA) components. *Right*: time-frequency (TF) representation (TFR) separately for the first and second sessions and for pain and touch. The outlined TF areas were selected to estimate the stability and variability of the gamma magnitude. The *bottom right* corner of each spectral plot contains the individual’s averaged rating of the sensory experience (scale 0 to 10). The topographies were normalized and are ranging from −1 to 1 (arbitrary units: a.u.).

**Figure 5.**
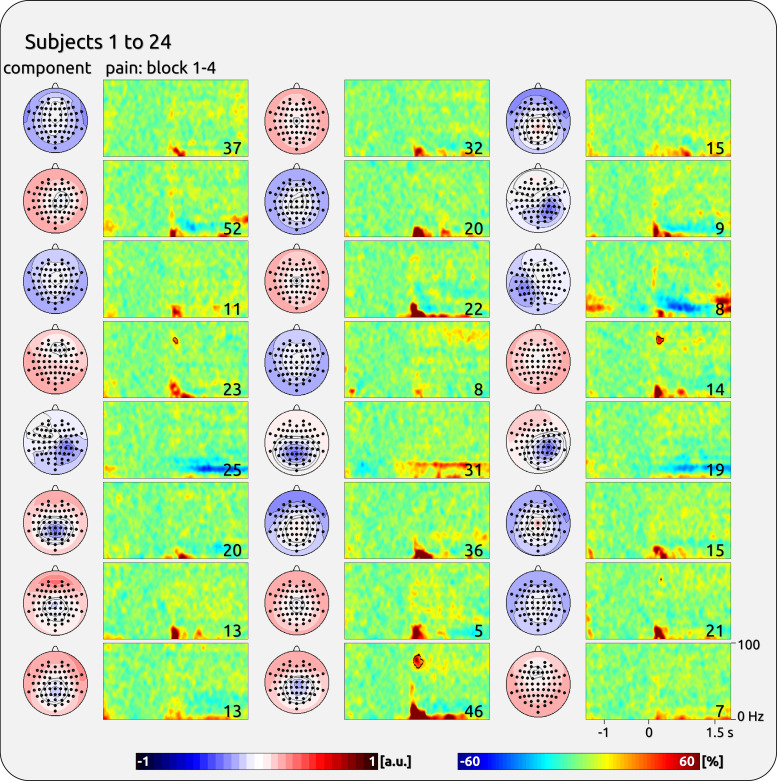
Single-subject components with gamma response from the first half of the participants of *dataset 2*. *Left*: topographies of independent component analysis (ICA) components. *Right*: time-frequency (TF) representation (TFR) plots. The outlined areas show the significant TF data. The *bottom right* corner of each spectral plot contains the individual’s average rating of the sensory experience (scale 0 to 100). The topographies were normalized and are ranging from −1 to 1 (arbitrary units: a.u.).

**Figure 6.**
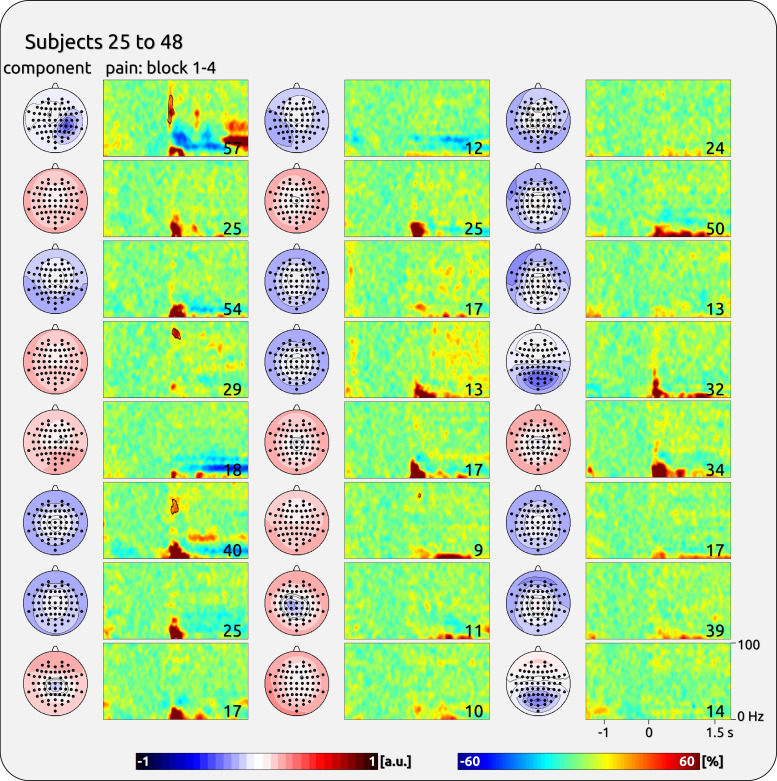
Single-subject components with gamma response from the second half of the participants of *dataset 2*. *Left*: topographies of independent component analysis (ICA) components. *Right*: time-frequency (TF) representation (TFR) plots. The outlined areas show significant TF data. The *bottom right* corner of each spectral plot contains the individual’s averaged rating of the sensory experience (scale 0 to 100). The topographies were normalized and are ranging from −1 to 1 (arbitrary units: a.u.).

The gamma responses show a broad variety across participants (Fig. 1, *C–E*). We have provided an exemplary impression from some participants (Fig. 2) as well as a complete overview of all participants’ responses to brief painful and tactile stimuli (Figs. 3, 4, 5, and 6).

### Individual Variability of Perception and Cortical Processing

#### Behavioral data.

The analysis shows the intensity of perceived pain is variable across subjects (Table 1). For *dataset 1*, there was significant variability of ratings across subjects for pain (*F* = 23.84, *P* < 0.001) and touch (*F* = 4.77, *P* < 0.001). The findings for pain were confirmed for *dataset 2* (*F* = 79.25, *P* < 0.001).

**Table 1.** Analysis of stability, session, subject variability, and dopamine depletion

*Dataset 1*	Pain	Pain γ ICA	Pain γ EEG	Touch	Touch γ ICA	Touch γ EEG
Session, 1st vs. 2nd	*F* = 2.16 (*P* = 0.16)	***F* = 17.46 **(*P* < 0.001)	*F* = 6.50 (FC6) (*P* = 0.21)	*F* = 1.03 (*P* = 0.32)	*F* = 2.56 (*P* = 0.13)	*F* = 2.05 (O1) (*P* = 0.95)
Dopamine depletion	*F* = 0.04 (*P* = 0.84)	*F* = 0.22 (*P* = 0.64)	*F* = 7.05 (PO3) (*P* = 0.10)	*F* = 0.46 (*P* = 0.50)	*F* = 0.01 (*P* = 0.94)	*F* = 2.05 (RE) (*P* = 0.93)
Stability subjects, 1st vs. 2nd	**τ = 0.73 **(*P* < 0.001)	**τ = 0.58 **(*P* < 0.001)	**τ = 0.71 (P1) **(*P* < 0.001)	**τ = 0.51 **(*P* = 0.001)	**τ = 0.65 **(*P* < 0.001)	**τ = 0.44 (CP1) **(*P* = 0.002)
Variability subjects	***F* = 23.84 **(*P* < 0.001)	***F* = 5.05 **(*P* < 0.001)	***F* = 14.72 (C1) **(*P* < 0.001)	***F* = 4.77 **(*P* < 0.001)	***F* = 7.23 **(*P* < 0.001)	***F* = 4.95 (FC1) **(*P* < 0.001)
*Dataset 2*: variability subjects	***F* = 79.25 **(*P* < 0.001)	***F* = 9.74 **(*P* < 0.001)	n/a	n/a	n/a	n/a

Analyses on multiarray EEG data were corrected for multiple testing using PALM. Bold entries indicate significant results. For EEG data, electrodes with the largest effect are shown. ICA, independent component analysis.

#### ICA data.

The magnitude of pain-related gamma oscillations was variable across subjects (Table 1). For *dataset 1*, there was a significant between-subject variability of induced gamma for pain (*F* = 5.05, *P* < 0.001) and touch (*F* = 7.23, *P* < 0.001). The findings for pain were confirmed for *dataset 2* (*F* = 9.74, *P* < 0.001). The interindividual variability is illustrated in Figs. 3, 4, 5, and 6; where individually significant gamma responses are outlined.

#### EEG data.

The analysis showed that the magnitude of stimulus-related gamma oscillations was variable across subjects (strongest effect at electrode C1: *F* = 14.72, *P* < 0.001; Table 1, Fig. 8) and touch (strongest effect at electrode FC1: *F* = 4.95, *P* < 0.001; Table 1, Fig. 8).

### Stability—Preserved Ranking of Participants

#### Behavioral data.

**Figure 7.**
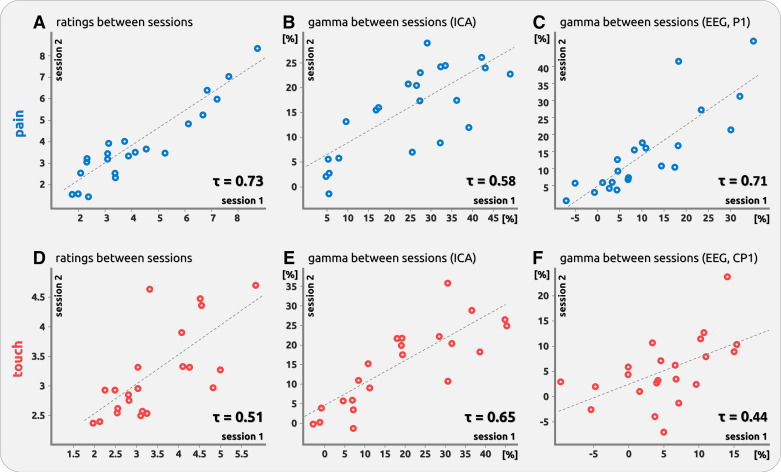
Scatterplots of individual ratings and evoked gamma in *dataset 1*. All scatter plots represent the stability measures across the two sessions of *dataset 1*. This applies to the pain domain (*top*; *A*, *B*, *C*), touch domain (*bottom*; *D*, *E*, *F*), as well as to averaged ratings (*left*; *A*, *D*), gamma derived from independent component analysis (ICA) components (*middle*; *B*, *E*), and gamma derived from EEG electrodes (*right*; *C*, *F*). Stability is expressed as Kendall’s τ. Data points for ICA and EEG data are shown as % signal change compared with the prestimulus baseline. Dashed least squares lines were included for illustration purpose. ICA, independent component analysis.

For *dataset 1*, we found consistent results of ratings between both experimental sessions. Subjects with high ratings in the first session had similarly high ratings in the second session; this applied to painful (τ = 0.73, *P* < 0.001; Table 1, Fig. 7*A*) and tactile stimulation (τ = 0.51, *P* = 0.001; Table 1, Fig. 7*D*).

#### ICA data.

We found consistent results of gamma magnitude between both experimental sessions. Subjects with high-magnitude gamma responses in the first session had similarly high magnitudes in the second session; this applied to both pain (τ = 0.58, *P* < 0.001; Table 1, Fig. 7*B*) and touch (τ = 0.65, *P* < 0.001; Table 1, Fig. 7*E*).

#### EEG data.

**Figure 8.**
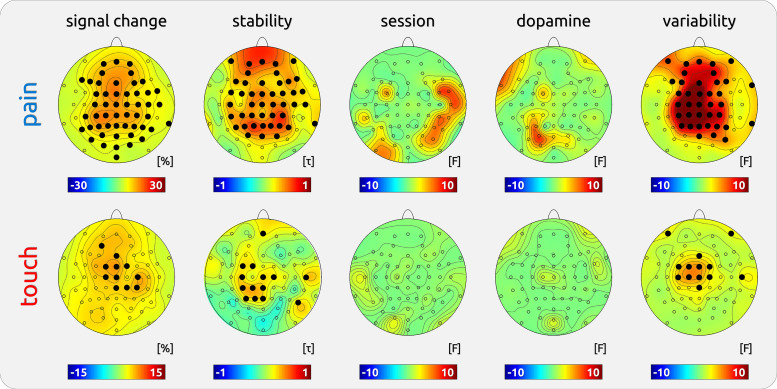
Topographies of experimental variables on neuronal gamma oscillations in *dataset 1*. Gamma responses were based on a predefined time-frequency window (150–350 ms; 76–86 Hz). Signal change: group effect of induced gamma responses to pain compared with the prestimulus baseline; Stability: Kendall’s τ correlation of gamma magnitude between two sessions; Session: effect of systematic signal change between first and second session; Dopamine: effect of dopamine depletion; Variability: occurrence of interindividual differences of gamma magnitudes. Solid dots indicate electrodes with significant effects (*P* < 0.05; corrected for multiple testing).

We found consistent results of gamma magnitudes between experimental sessions. Subjects with a high gamma magnitude in the first session had similar magnitude in the second session for painful (strongest effect at electrode P1: τ = 0.71, *P* < 0.001; Table 1, Figs. 7*C* and 8) and tactile stimulation (strongest effect at electrode CP1: τ = 0.44, *P* = 0.002; Table 1, Figs. 7*F* and 8). The effect was mainly located at vertex electrode sites for both modalities.

### Stability—Systematic Change between Sessions

#### Behavioral data.

Ratings of pain and touch were not affected by the order of the experimental sessions (pain: *F* = 2.16, *P* = 0.16; touch: *F* = 1.03 *P* = 0.32, Table 1). There was no effect of dopamine depletion on pain ratings (*F* = 0.04, *P* = 0.84, Table 1) and touch ratings (*F* = 0.46, *P* = 0.50, Table 1).

#### ICA data.

Gamma magnitudes were not affected by the order of the experimental sessions for touch (*F* = 2.56, *P* = 0.13) but were for pain (*F* = 17.46, *P* < 0.001). Independent of the modulation of dopamine depletion, the first pain session exhibited a higher gamma magnitude than the second session (Table 1). There was no significant effect of dopamine depletion for either pain (*F* = 0.22, *P* = 0.64) or touch (*F* = 0.01, *P* = 0.94, all Table 1).

#### EEG data.

There was no significant effect of the order of stimulation on gamma responses across sessions as recorded at scalp vertex for both pain (electrode FC6: *F* = 6.50, *P* = 0.21; Table 1, Fig. 8) and touch (electrode O1: *F* = 2.05, *P* = 0.95; Table 1, Fig. 8). There was no significant effect of dopamine depletion on gamma responses for both pain (electrode PO3: *F* = 7.05 *P* = 0.10; Table 1, Fig. 8) and touch (right eye electrode RE: *F* = 2.05, *P* = 0.93; Table 1, Fig. 8).

### Stability—Similarity of Time-Frequency Patterns across Sessions

#### ICA data.

**Figure 9.**
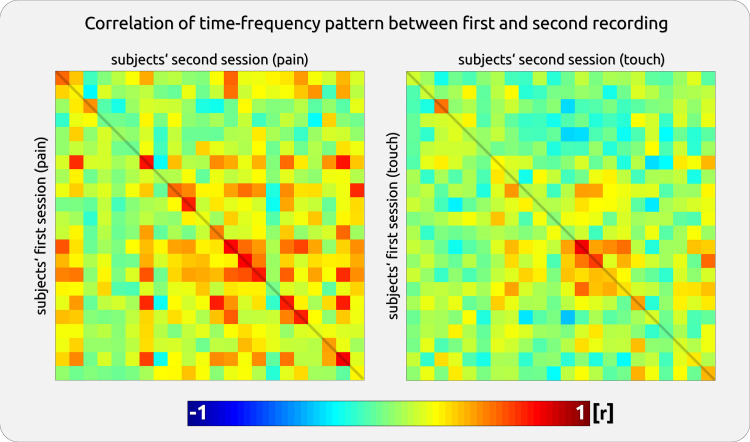
Stability of gamma patterns across sessions. For *dataset 1*, we computed (*left*: pain, *right*: touch) a point-by-point correlation between the gamma time-frequency pattern (0–500 ms/40–100 Hz) from the selected components. The first session of a subject was correlated with their own (diagonal) and all other subjects’ time-frequency patterns (extra-diagonal). Each of the 22 rows/columns represents a single subject. The highest correlation was usually found within a subject (the diagonal). A high within-subject correlation can only be obtained for participants with high gamma magnitudes but not for subjects with low gamma (e.g., *subject 4*).

The analysis is based on the individual participants’ TF plots across repeated sessions (Fig. 2, *A* and *B*). The results of the similarity analysis showed a stronger coefficient for the within-subject correlation between sessions (the diagonal in Fig. 9) compared with the correlation for the subjects’ first recordings with the averaged correlations of the second recordings of the other subjects (correlation coefficients outside the diagonal). This applied to gamma responses to pain (paired *t* test, *t* = 4.91, *P* < 0.001) and touch (paired *t* test, *t* = 4.03, *P* < 0.001).

## DISCUSSION

Here, we aimed to investigate the interindividual variability and the individual stability of induced EEG gamma oscillations to phasic noxious laser stimuli and innocuous tactile stimuli. The results showed a large individual topographical variability of the gamma responses, indicating a variety of underlying gamma sources across individuals. Hence, due to the different locations of the gamma responses across the scalp, this variety cannot be taken into account by electrode/sensor-level analyses. For this reason, we are resting our interpretations mainly on the findings from ICA data.

Using two datasets we showed a broad variety of high, low, and no gamma responses across participants. For one dataset with repeated sessions, we were able to provide complementary descriptors of individual stability of gamma oscillations by integrating the information from three statistical approaches, i.e., *1*) a magnitude correlation across sessions, *2*) the effect of magnitude change across sessions, and *3*) the analysis of the similarity of the TF patterns across sessions. Overall, the individual expression of gamma oscillations was remarkably stable. For example, we observed that participants with a high gamma magnitude in the first session also exhibited a high gamma magnitude and a similar gamma TF pattern in the subsequent session.

### Interindividual Variability of Cortical Gamma Oscillations

The present study was sparked by previous study observations (5, 11, 30) that some participants do not mirror the group results, and instead lack gamma response to laser stimulation. Here, we have quantified the broad variety of neuronal gamma responses across subjects in terms of magnitude, TF distribution, and topography. Our regression model addressed the variability of individual gamma magnitudes by revealing a significant factor “subject,” which is suggested to be unrelated to a subject’s sensitivity to painful or tactile stimulation (see *Methodological Consideration*). This is illustrated by the individual TF plots depicting a clear and significant gamma response in some subjects and an absence thereof in others. In a similar vein, the variability and uniqueness of individual gamma patterns are represented by the high correlation of the TF representation between the two sessions of a single participant; this individual gamma pattern is significantly different from the patterns of the other participants. Consequently, individual TF patterns can reliably extend beyond the repeatedly reported group effect around 80 Hz and 250 ms (5, 10, 11, 36). In addition, while some participants did not show a gamma response on any component, others exhibited gamma on more than one ICA component. Furthermore, there is also some variation regarding the topographical distribution of individual gamma responses, represented by an interindividual diversity of distributions and local maxima at lateral, fronto-central, or parietal electrodes.

### Individual Stability of Cortical Gamma Oscillations

At group level, stability and reliability of EEG parameters (including gamma oscillations) have been investigated before (42–44), yet to date, no study has provided evidence on the stability of induced gamma synchronization. Here, the stability of the experimental effects across sessions offers a mixed picture derived from complementary analyses.

We show remarkable stability of individual gamma responses and somatosensory ratings indicated by a similar ranking of the participants across sessions. Participants with high gamma activity and high pain/touch ratings in the first session had high gamma activity and high pain/touch ratings in the second session after 2 wk. The same applies to participants with low or no gamma and low pain/touch ratings. These findings are accompanied by a similarity of the TF patterns for gamma responders across sessions.

Furthermore, there is no overall change in ratings and gamma magnitude at electrode level, but a significant effect of change for the magnitude of pain-induced gamma oscillations for ICA data. Overall, participants exhibited a higher gamma magnitude in the first session compared with the second session. The significant effect of pain magnitude change for ICA data indicates the higher precision of our approach using individually adapted TF windows from a variety of different gamma topographies. The disadvantage of the analyses at electrode level can be attributed to the mixture of EEG signals receiving information from multiple underlying cortical sources. Conversely, the different ICA gamma topographies suggest that gamma can be individually dispersed to distinct electrodes. Furthermore, a universal and predefined TF window will neglect important information from outside the window.

Taken together, the significant effect of change across sessions for ICA-transformed data but not for electrode-level data supports the idea of individual gamma processing in distinct cortical regions giving rise to different cortical gamma maps and individually unique gamma response patterns.

### Emphasis on Reproducibility Neglects Individual Differences

Previous research suggests there is converging evidence that group results on gamma oscillations might be replicable across studies with different samples. Indeed, gamma responses to laser pain have been reported by a number of studies (3, 9, 45), however, a simple repetition of findings at group level for pain- and touch-induced gamma will be inevitably misleading for a number of reasons:

First, group studies do not sufficiently take into account the variability of gamma across individuals. For example, the interpretation of group findings implies that each and every participant shows a gamma response at around 80 Hz (5, 38). Studies imply or explicitly state that nociceptive-related gamma oscillations reflect the most important mechanism for encoding the subjective pain perception in each and every participant (11, 18, 19, 38). Our current findings in two different datasets, where only some individuals display robust and consistent gamma responses, provide a more nuanced view.

Second, our findings suggest the notion of multiple gamma response patterns, characterized by a variety of topographical and TF distributions for both somatosensory modalities. This suggests that group analyses will eventually fail to adequately reflect the processing of pain and touch in a large subset of the samples, especially in the absence of gamma. Consequently, studies may overestimate the contribution of a cortical process (i.e., gamma oscillations) to pain and touch perception and make inaccurate inferences about the somatosensory processing mechanisms in the population. The only study that reported a TF variability of gamma responses would have benefited from repeated recordings to confirm the stability of individually unique activity patterns (17).

Third, although group statistics reported a positive within-subject correlation between the magnitude of gamma oscillations and pain intensity, single-subject data also showed a negative correlation (6, 14). The variability of ICA maps further substantiates the notion that the results of group statistics do not necessarily reflect the cortical processing of the individual (46, 47). We suggest future studies to adopt multiple repeated recordings of the same participants to shed light on this widely neglected issue.

### Methodological Considerations

Traditionally, the between-subjects variability we investigated here is mostly treated as noise. In two separate datasets, we observed a large variability of somatosensory gamma responses with different topographies, individual TF windows in some participants, and a complete absence of any gamma response in others. Unfortunately, our findings cannot offer any explanation about the underlying mechanisms of the interindividual variability of gamma. In the pain domain, there are contradictory results on the dependency of gamma magnitudes on a subject’s sensitivity (11, 31). However, this type of analysis is problematic because it is based on electrode-level statistics; the present findings suggest the existence of individual gamma sources as reflected by different topographical distributions. A correlation between behavioral data and activities from potentially different sources does not appear to be appropriate. Instead, our results suggest no relationship between the occurrence of gamma and a subject’s sensitivity. Both datasets include participants with high pain ratings and no gamma response, thus shedding light on the complexity of cortical processing.

Furthermore, we did not take the complete information on gamma into account, as we have limited the analysis of ICA data to a maximum of one component per participant. In fact, as some participants exhibited more than one gamma component, it would have been equally reasonable to include all components showing a significant gamma response. However, this would have resulted in an unequal number of components across participants.

Please note that the analysis on the similarity of the TF patterns across sessions may underestimate the “real” stability of these patterns, as subjects with no gamma naturally exhibit low correlations of the TF patterns (the green squares in the diagonal in Fig. 9). The evaluation of the statistical analysis on similarity should be accompanied by a visual inspection of the individual gamma patterns across sessions.

Finally, there is a debate on whether it is appropriate to calibrate the applied stimulus intensity to the perception of the individual (e.g., to a subjective 5 of 10). A recent publication discusses the advantages and disadvantages of this approach (48). For the present publication, a calibration would have benefitted the analysis of within-subject variability as all participants would have centered their ratings around the same value (e.g., a subjective 5 of 10). In contrast, a calibration to e.g., a subjective rating of 5 for all participants would have made it impossible to analyze the stability of ratings between sessions, as the computation of Kendall’s τ relies on the variability across individuals.

### Conclusions

Overall, the results show a remarkable variability of gamma oscillations across individuals, but also remarkable stability of these responses within the individual. These findings have important implications for the generalizability of the functional significance of gamma oscillations in the context of pain and touch. Indeed, both the variability and stability of oscillatory patterns have been largely neglected in previous studies that focused on group statistics. Future research may aim to elucidate the conditions under which some subjects have or have not developed somatosensory gamma oscillations. It also remains to be seen whether some individuals would display stable cortical processes during long-lasting tonic pain or in chronic pain conditions. The same line of questioning on somatosensory gamma oscillations may apply to other modalities (e.g., vision) and cortical oscillations (e.g., α band).

In conclusion, the current results support the notion of interindividual variability and intraindividual stability of event-related neural gamma synchronization and argue for a greater emphasis on subject-level assessment of sensory responses, particularly in the context of pain perception.

## DATA AVAILABILITY

The data from study 1 can not made available due to the missing permission from the participants. The data from study 2 is available at the Open Science Framework (https://osf.io/jw8rv/).

## SUPPLEMENTAL DATA

10.6084/m9.figshare.19740121 Supplemental Material: https://doi.org/10.6084/m9.figshare.19740121.

## DISCLOSURES

No conflicts of interest, financial or otherwise, are declared by the authors.

## AUTHOR CONTRIBUTIONS

E.S. conceived and designed research; E.S. performed experiments; A.Shindy, V.W., and E.S. analyzed data; E.V., A.Stankewitz, and E.S. interpreted results of experiments; E.S. prepared figures; E.V., A.Shindy, V.W., A.Stankewitz and E.S. drafted manuscript; E.V., A.Stankewitz, and E.S. edited and revised manuscript; E.V., A.Shindy, V.W., A.Stankewitz, and E.S. approved final version of manuscript.
